# Linking Pre-Diabetes with Benign Prostate Hyperplasia. IGFBP-3: A Conductor of Benign Prostate Hyperplasia Development Orchestra?

**DOI:** 10.1371/journal.pone.0081411

**Published:** 2013-12-19

**Authors:** Ioannis Protopsaltis, Achilles Ploumidis, Theodoros N. Sergentanis, Padelis Constantoulakis, Kostantinos Tzirogiannis, Chrysoula Kyprianidou, Athanasia K. Papazafiropoulou, Andreas Melidonis, Dimitrios Delakas

**Affiliations:** 1 Diabetes Center, Tzanio General Hospital of Piraeus, Piraeus, Greece; 2 Department of Urology, Asklipieio General Hospital, Voula, Greece; 3 Department of Hygiene, Epidemiology and Medical Statistics, Medical School, National University of Athens, Athens, Greece; 4 Department of Molecular Pathology and Genetics, Locus Medicus Laboratory, Athens, Greece; 5 3rd Department of Internal Medicine and Center of Diabetes, General Hospital of Nikaia “Ag. Panteleimon”, Piraeus, Greece; Louisiana State University Health Sciences center, United States of America

## Abstract

Benign prostatic hyperplasia (BPH) represents a pattern of non-malignant growth of prostatic fibromuscular stroma. Metabolic disturbances such us pre-diabetes and metabolic syndrome may have a role in BPH pathophysiology. A potential explanation for the above relationship involves the insulin-like growth factor (IGF) axis as well as IGF binding proteins, (IGFBPs) of which the most abundant form is IGFBP-3. Therefore, the aim of the present study was to investigate the association between intra-prostatic levels of IGF-1, IGF-2 as well as to evaluate the role of locally expressed IGFBP-3 in BPH development in pre-diabetes. A total of 49 patients admitted to the Urology department of a tertiary urban Greek hospital, for transurethral prostate resection, or prostatectomy and with pre-diabetes [impaired fasting glucose (IFG) and impaired glucose tolerance (IGT) or both] were finally included. The majority of the sample consisted of subjects with IGT (51.0%), followed by IFG and IGT (32.7%) and isolated IFG (16.3%). For all participants a clinical examination was performed and blood samples were collected. In addition, total prostate (TP) volume or transitional zone (TZ) volume were estimated by transrectal ultrasonography. The results of the multivariate analysis regarding TP volume showed that higher PSA (p<0.001), larger waist circumference (p=0.007) and higher IGFBP-3 expression levels (p<0.001) independently predicted higher TP volume. The results regarding the volume of the TZ showed that higher PSA (p<0.001), larger waist circumference (p<0.001) and higher IGFBP-3 expression levels (p=0.024) were independently associated with higher TZ volume. Our findings show that intra-prostatic levels of IGFBP-3, PSA and waist circumference, but not overall obesity, are positively associated with prostate volume. IGFBP-3 seems to be a multifunctional protein, which can potentiate or inhibit IGF activity.

## Introduction

Benign prostatic hyperplasia (BPH) represents a pattern of unregulated but non-malignant growth of prostatic fibromuscular stroma [1]. Although there is evidence that ageing and hormonal alterations are involved in growth of stromal and epithelial components in the prostate and induction of fibromuscular overgrowth, the pathogenesis of BPH remains still unclear [2,3]. BPH pathogenesis seems to be multifactorial and recent findings highlight the key role of metabolic disturbances such us obesity, disturbances of glucose homeostasis and metabolic syndrome (MS) in BPH pathophysiology [2,3]. 

Metabolic syndrome (MS) is a clinical syndrome, easily identified, that predisposes to an increased risk of developing benign prostatic hypertrophy. Hamarsten et al. [4], have reported that men with components of MS had significantly larger prostate volumes and BPH growth rates. In this context Nandeesha et al. [5], have also reported that fasting serum insulin was significantly higher in men with BPH than in controls without BPH and obesity, elevated fasting plasma glucose levels, diabetes, were risk factors for developing benign prostatic hyperplasia [6].

Insulin resistance (IR), apart from changes in carbohydrate, lipid, or protein metabolism, affectes growth, differentiation, DNA synthesis, regulation of gene expression and BPH [7]. A potential explanation for the association of BPH with hyperinsulinemia, involves the insulin-like growth factor (IGF) axis. IGF-1 and IGF-2 are peptides produced by prostatic cells, critical in the regulation, development, and proliferation of prostatic stroma cells and elevated serum concentrations of insulin and IGF-1 have been associated with BPH [8,9]. Hyperinsulinemia further stimulates IGF-1 production by upregulating growth hormone (GH) receptors in the liver [10]. It is known that GH stimulates IGF production by the liver [10].

IGFs are transported in the circulation bound to their carrier proteins; IGF binding proteins, (IGFBPs) of which the most abundant form is IGFBP-3, which has also been associated with prostatic growth and insulin [11,12]. However, data on the association of IGFBP-3 and BPH development are conflicting with some researchers to report that elevated IGFBP-3 concentrations correlate with increased BPH risk [3], whereas others have reported an inverse correlation [13,14]. 

In the majority of studies examining the relationship of IGF axis and various anthropometric variables with BPH, only circulating levels of IGFs or IGFBPs, and not prostate tissue levels were examined. However, local tissue expression of IGFs and IGFBPs could be more important and accurate in evaluating these associations, since the total circulating pool of the above factors may not reflect intra-prostatic levels or biological activity [15,16]. 

IR and obesity are part of the clinical entity characterized as pre-diabetes [17] which represents a heterogeneous group of metabolic defects preceding type 2 diabetes (T2D) [18]. Prediabetes encompasses impaired fasting glucose (IFG) and impaired glucose tolerance (IGT) states, with both to be characterized by IR [19]. In addition IGT has been reported to be accompanied by higher IR levels than IGF [20] by some researchers. Both IFG and IGT represent deranged glucose homeostasis states that significantly increase risk of progression to frank T2D especially when in coexistence [18]. 

The aim of the present study was to investigate the association between intra-prostatic levels of IGF-1, IGF-2 and BPH, as well as to evaluate the role of locally expressed IGFBP-3 in BPH development in different states of pre-diabetes. 

## Materials and Methods

### Ethics statement

All participants gave written informed consent. The study was approved by the ethics committee of the Tzanio General Hospital of Piraeus, Greece and followed the ethical standards of the Helsinki Declaration.

Patients admitted to the Urology department of a tertiary urban Greek hospital, for transurethral prostate resection (TURP), or prostatectomy comprised the initial study population. Patients with a previous history of malignancy, inflammatory disorders, prostate surgery, and diabetes were excluded. Subjects treated with α-blockers, 5-a reductase inhibitors and/or metformin were also excluded. A total of 49 patients with histologically proven BPH and not prostate cancer, based on biopsy of prostate tissue removed during these procedures, and with pre-diabetes (IGF, IGT, or both) were finally included. Therefore all participants underwent a 75gr oral glucose tolerance test (OGTT) for determining glucose tolerance status according to the criteria of the American Diabetes Association and only individuals with pre-diabetes were enrolled. IFG diagnosis was based on fasting plasma glucose (FPG) value > 100 mg/dL and < 126 mg/dL. Diagnosis of IGT was based on serum glucose concentration 2-h PG > 140 mg/dL and < 200 mg/dL. 

For all participants the following parameters were determined at baseline by trained interviewers: age, body mass index (BMI, body weight kilograms divided by the square of height in meters), waist to hip ratio (waist circumference divided by hip circumference), systolic blood pressure (SBP) and diastolic blood pressure (DBP), and MS status (according to the criteria set by the National Cholesterol Education Program). In addition the cumulative number of MS components was also determined. Blood samples were drawn and biochemical analyses including serum glucose, total cholesterol, low density lipoprotein cholesterol (LDL-C), triglycerides, and high density lipoprotein cholesterol (HDL-C). Total prostate specific antigen (PSA) levels were also determined. 

Total prostate (TP) volume or transitional zone (TZ) volume, estimated by transrectal ultrasonography, and were used as surrogate measures of degree of BPH. All patients underwent transrectal ultrasound before operation for calculation of TP and TZ volumes using the ellipsoid method. All TRUS were performed by the same urologist. Intra-prostatic expression of IGF-1, IGF-2, and IGFBP-3 were evaluated with RT-PCR as described below. 

### RNA Extraction from Tissues

RNA from the tissues treated with Allprotect Tissue reagent (QIAGEN, Cat. # 76405) was extracted with the use of the QIAGEN AllPrep® DNA/RNA/Protein Mini kit (Cat. # 80004). Briefly, approximately 20 mg of tissue was disrupted using the mortar and pestle method described in the QIAGEN AllPrep® DNA/RNA/Protein Mini kit handbook. Homogenization of the disrupted tissue was obtained by the use of QIAshredder homogenizer. Total RNA, DNA and protein was obtained as described at the QIAGEN AllPrep® DNA/RNA/Protein Mini kit handbook. The RNA elute (30 μl in total) was used for the real-time PCR quantification of IGF-1, IGF-2 and IGFBP-3.

### Real-Time PCR primers and probes

Primers specific for the amplification of each of the IGF-1, IGF-2 and IGFBP-3 genes, as well as for the reference gene β2-microglobulin were ordered for synthesis at TIB MOLBIOL. Hybridization probes suitable for each gene were constructed by TIB MOLBIOL. Forward primer for IGF-1: 5’-TgTgTggAgACAggggCTT-3’, reverse primer for IGF-1: 5’-TgCgTTCTTCAAATgTACTTCCTT-3’. Forward primer for IGF-2: gACACCCTCCAgTTCgTCTg, reverse primer for IGF-2: CggggTATCTggggAAgTTgT. Forward primer for IGFBP-3: TCTCAgAgCACAgATACCCAgAAC, reverse primer IGFBP-3: ggAAgggCgACACTgCTTT-3’. Forward primer β2-microglobulin: CCAgCAgAgAATggAAAgTC, reverse primer β2-microglobulin: gATgCTgCTTACATgTCTCg. Hybridization probes IGF-1: 5’-TgTATTgCgCACCCCTCAAgCC—FL and CCAAgTCAgCTCgCTCTgTCCgT—PH. Hybridization probes IGF-2: CCgTggCATgTTgAggAgTgCT—FL and TTTCCgCAgCTgTgACCTggCC—PH. Hybridization probes IGFBP-3: CTCAATgTgCTgAgTCCCAggggTgT—FL and CACATTCCCAACTgTgACAAgAAgggA—PH. Hybridization probes β2-microglobulin: TTCTTCAgTAAgTCAACTTCAATgTCggA—FL and ATgAAACCCAgACACATAgCAATTCAg—PH. 

### Real-Time PCR conditions

For each reaction, the following PCR mix was prepared: 7.9 μl dH2O, 1.3 μl Mn(OAc)2 (3.25 mM), forward and reverse primers 0.2 μl (0.2 μM), 0.2 μl (0.1 μM) of each probe and 7.5 μl LightCycler RNA Master HybProbe (Cat. # 03018954001). 17.5 μl of the pcr mix and 2.5 μl of RNA was added into a glass capillary to a total volume of 20 μl. Briefly, the One-Step RT PCR conditions used for IGF-1, IGF-2 and IGFBP-3 were the following: RT (20 min, 61°C), Initial Denaturation (1 min, 95 °C), amplification and quantification program (95 °C, 3 sec; 48 °C, 12 sec; 72° C, 10 sec; these steps were repeated for 50 cycles), melting program (95° C, 10 sec; 45 °C, 30 sec;85 °C, 0 sec) and cooling to 40 °C. For the β2-microglobulin One-Step Real-Time PCR the following conditions were used: RT (20 min, 61°C), Initial Denaturation (1.0 min, 95 °C), for the PCR (95 °C, 3 sec; 48 °C, 12 sec; 72 °C, 10 sec; these steps were repeated for 50 cycles), melting program (95 °C, 0sec; 46 °C, 30 sec; 80 °C, 0 sec) and cooling to 40 °C. The experiment was repeated twice using the ROCHE LightCycler 1.5 instrument. 

### Quantification values

The relative gene expression was estimated by incorporating the crossing point of each sample for each of IGF-1, IGF-2 and IGFBP-3, as well as the crossing point for β2-microglobulin to the following formula: 2(ΔCt sample-ΔCt calibrator). All real-time PCR expression data are presented as arbitrary units (AU)[21,22]. 

### Statistical analysis

 For the evaluation of the factors associated with total prostate volume, as well as transitional zone volume, a standard two-step approach was followed: univariate and multivariate analysis. At the univariate analysis, parametric tests were appropriately implemented after the log-transformation of total prostate volume and the volume of the transitional zone, given that the log-transformed volumes followed the normal distribution (as attested by the Shapiro-Wilk test). 

The factors whose associations with TP and TZ volume were examined comprised the following: age, BMI, waist circumference, hip circumference, WHR, subclassification of pre-diabetes (IFG, IGT, IFG+IGT), IGF-1 levels, IGF-2 levels, IGFBP-3 levels, PSA, MS, SBP, total cholesterol, LDL-C, HDL-C, and triglycerides.

At the multivariate analysis, stepwise linear regression was performed. As appropriate, factors proven significant at the univariate analysis were tested in the stepwise multivariate model as independent variables; a subset of them (i.e., those with p<0.05) were appropriately retained during the stepwise selection of variables. In case of conceptually intertwined variables (such as waist circumference and WHR), alternative models were constructed. Normality of the studentized (jackknifed) residuals was verified using the Shapiro-Wilk test for each model. Data are expressed as mean ± standard deviation unless it is stated elsewhere. Statistical analysis was performed using STATA 11.1 statistical software (Stata Corporation, College Station, TX, USA). 

## Results

### Study population

**Figure 1:**
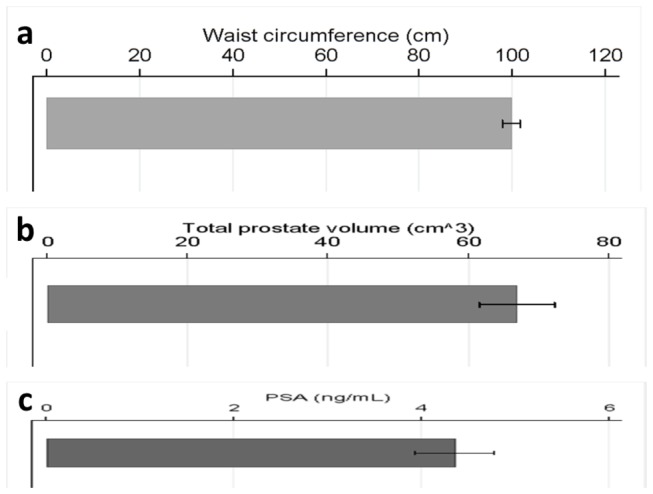
(a) Waist circumference, (b) total prostate size and (c) PSA values (mean±standard error) in the study sample.

Table 1 presents the description of the study sample. The mean age was 71.3±7.3 years (range: 62-88 years); the majority (n=25) of the sample consisted of subjects with IGT (51.0%), followed by IFG and IGT 16 (32.7%) and isolated IFG 8 (16.3%). 51% (n=25) of prediabetic subjects had MS according to the NCEP ATP III criteria. The total prostate volume was 66.9±37.2 cm^3^, whereas the transitional zone volume was 44.8±31.1 cm^3^. Figure 1 graphically presents waist circumference, total prostate size and PSA values in the study sample. 

**Table 1:** Demographic and clinical characteristics of the study population.

	**Value**
Age (years)	71.7±7.3
Total prostate volume (cm^3^)	66.9±37.2
Transitional zone volume (cm^3^)	44.8±31.1
PSA (ng/mL)	4.36±2.92
IGF-1 (AU)	0.106±0.100
IGF-2 (AU)	0.064±0.124
IGFBP-3 (AU)	0.048±0.082
BMI (kg/m^2^)	27.0±2.3
Waist circumference (cm)	99.9±13.1
Hip circumference (cm)	98.2±10.9
WHR	1.02±0.05
Total Cholesterol (mg/dL)	195.3±42.9
HDL-C (mg/dL)	46.9±12.4
LDL-C (mg/dL)	126.7±47.9
Tg (mg/dL)	86.7±32.1
SBP (mmHg)	132.5±16.2
Metabolic syndrome (Yes) n (%)	25 (51.0)
IFG (Yes) n (%)	8 (16.3)
IGT (Yes) n (%)	25 (51.0)
IFG and IGT (Yes) n (%)	16 (32.7)

Note: PSA, prostate specific antigen; IGF, insulin-like growth factor; IGFBP-3, insulin-like growth factor binding protein-3; BMI, body mass index; WHR, waist to hip ratio; HDL-C, high density lipoprotein cholesterol; LDL-C, low density lipoprotein cholesterol; Tg, triglycerides; SBP, systolic blood pressure; IFG impaired fasting glucose; IGT, impaired glucose tolerance.

### Univariate Analysis

**Figure 2:**
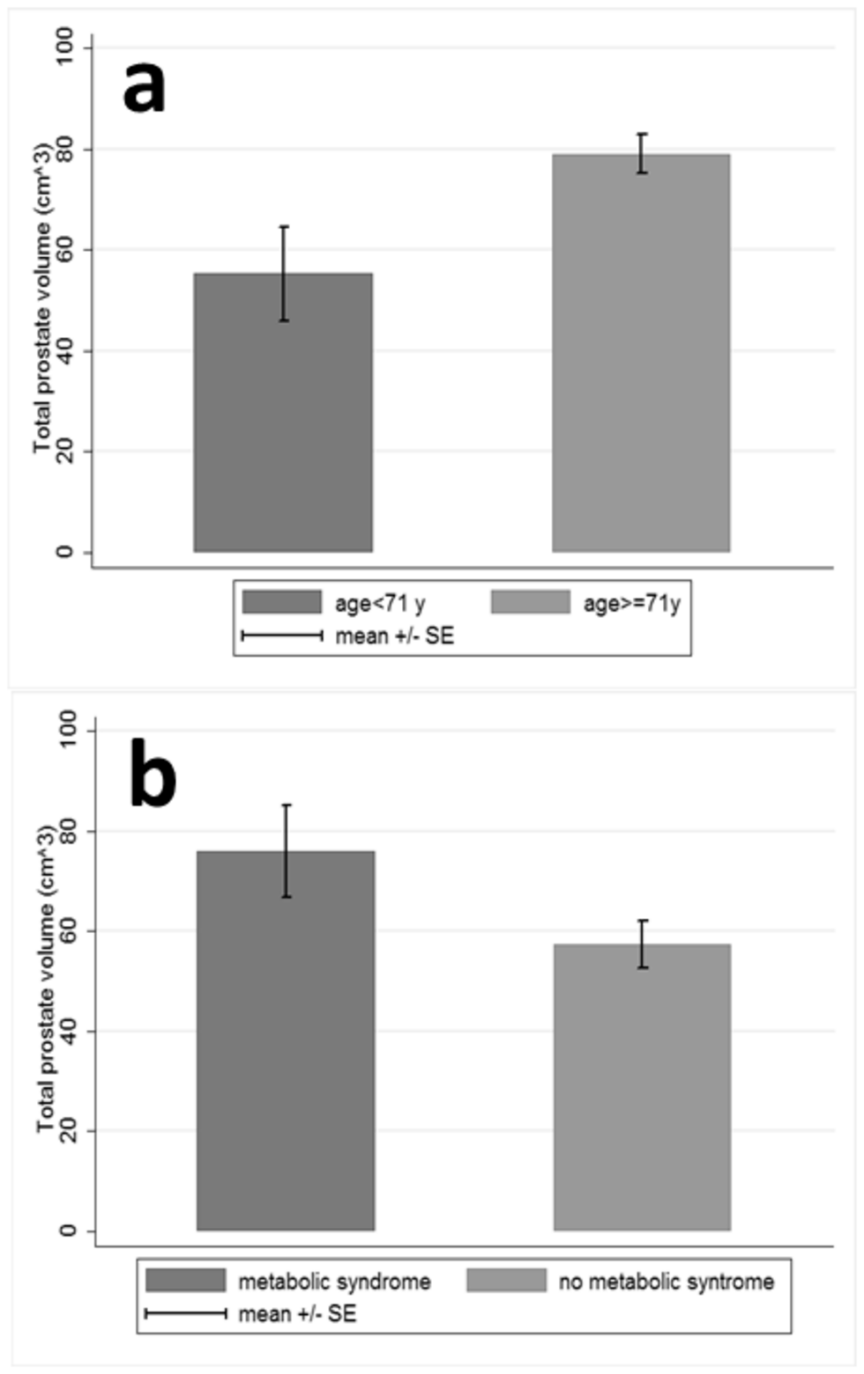
Total prostate volume (mean±standard error) in (a) younger versus older subjects, (b) subjects with versus without metabolic syndrome.

Table 2 presents the variables that were significantly associated with TP volume and/or volume of the TZ at the univariate analysis. Larger TP volume was associated with higher PSA levels (r=+0.880, p<0.0001), higher IGF-2 expression levels (p=0.023), higher IGFBP-3 expression (p<0.0001), older age (p=0.004, Figure 2a), larger BMI (p=0.042), larger waist circumference (p=0.0001), hip circumference (p=0.027), WHR (p<0.0001), higher LDL-C (p=0.010) and triglyceride levels (p=0.016). TZ volume presented with a similar pattern of associations, except for the fact that age (p=0.400), BMI (p=0.118) and LDL-C (p=0.141) did not reach significance.

**Table 2:** Results of the univariate analysis.

**Continuous Variables**	**total prostate volume (mean±SD)**	**Pearson’s r**	**p-value**	**transitional zone volume (mean±SD)**	**Pearson’s r**	**p-value**
**PSA (ng/mL)**						
<4.16	44.6±9.9	**+0.880**	**<0.0001**	27.7±13.5	**+0.881**	**<0.0001**
≥4.16	100.0±42.8			64.2±34.9		
**IGF-2 (AU)**						
<0.031	48.3±18.6	**+0.325**	**0.023**	29.0±14.8	**+0.366**	**0.026**
≥0.031	84.7±42.0			52.4±34.1		
**IGFBP-3 (AU)**						
<0.026	59.7±24.0	**+0.588**	**<0.0001**	39.2±22.1	**+0.471**	**0.003**
≥0.026	73.7±46.0			49.2±36.5		
**Age(years)**						
<71	55.2±45.9	**+0.401**	**0.004**	44.3±47.7	+0.143	0.400
≥71	79.0±19.8			45.1±18.0		
**BMI (kg/m^2^)**						
<27.0	61.9±47.1	**+0.292**	**0.042**	42.8±16.0	+0.262	0.118
≥27.0	71.6±24.4			49.2±50.8		
**Waist Circumference (cm)**						
<103	57.2±26.5	**+0.544**	**0.0001**	34.9±24.6	**+0.728**	**<0.0001**
≥103	74.4±45.6			57.3±36.1		
**Hip Circumference (cm)**						
< 101	56.0±24.2	**+0.331**	**0.027**	38.5±23.1	**+0.550**	**0.0009**
≥ 101	79.0±48.5			58.6±41.5		
**WHR**						
<1.01	45.9±17.2	**+0.789**	**<0.0001**	27.6±15.5	**+0.842**	**<0.0001**
≥1.01	90.5±43.2			64.2±34.9		
**LDL-C (mg/dL)**						
<108	53.7±50.3	**+0.364**	**0.010**	36.7±50.0	+0.247	0.141
≥108	76.7±18.7			49.2±12.4		
**Tg (mg/dL)**						
<85	62.5±49.3	**+0.342**	**0.016**	38.6±43.4	**+0.464**	**0.004**
≥85	70.1±25.2			50.2±13.5		

Variables significantly associated with total prostate volume and/or volume of the transitional zone. Continuous variables have been presented as <median and median for purely descriptive reasons; their continuous nature has been appropriately taken into account at the univariate tests.

Note: PSA, prostate specific antigen; IGF, insulin-like growth factor; IGFBP-3, insulin-like growth factor binding protein-3; BMI, body mass index; WHR, waist to hip ratio; LDL-C, low density lipoprotein cholesterol; Tg, triglycerides.

Of note, IGF-1 expression levels were not associated with TP volume (r=+0.189, p=0.198) or with TZ volume (r=-0.095, p=0.583). Similarly, prediabetes subgroup was not associated with TP volume (IFG group: 60.3±15.8 cm^3^, IGT group: 73.1±45.4 cm^3^, IFG+IGT group: 60.5±29.9 cm^3^; F=0.64, p=0.530, ANOVA) or with TZ volume (IFG group: 30.1±16.0 cm^3^, IGT group: 50.8±38.3 cm^3^, IFG+IGT group 44.1.5±13.3 cm^3^, F=1.49, p=0.240, ANOVA). 

Non-significant trends were observed towards a positive association between MS and higher TP volume (76.0±45.1 cm^3^ for subjects with MS vs. 57.3±24.1 cm^3^ for those without, p=0.079, Student’s t-test, Figure 2b), as well as higher TZ volume (53.3.±37.3 cm^3^ for subjects with MS vs. 38.0±22.0 cm^3^ for those without, p=0.089, Student’s t-test). SBP was not associated either with TP or with TZ volume. In accordance with the positive association between higher LDL-C and TP volume, total cholesterol exhibited a trend of marginal significance towards positive association with the latter (r=+0.263, p=0.069), whereas HDL-C pointed to the opposite direction, as expected (r=-0.277, p=0.054).

### Multivariate analysis

Table 3 presents the results of the stepwise multivariate linear regression analysis regarding TP volume. Higher PSA (coefficient: +0.111, 95%CI: +0.090 to +0.133, p<0.001), larger waist circumference (coefficient: +0.008, 95%CI: +0.002 to +0.013, p=0.007) and higher IGFBP-3 expression levels (coefficient: +1.652, 95%CI: +0.989 to +2.314, p<0.001) independently predicted higher TP volume; of note, BMI lost its significance at the multivariate analysis, as evident from the aforementioned model. The alternative model, at which WHR was entered instead of waist circumference at the stepwise algorithm, replicated the results of the aforementioned model.

**Table 3:** Results of the stepwise multivariate linear regression analysis.

**Variables Category or Increment**	**Coefficient (95% confidence interval)**	**P-value**
PSA (1 ng/mL increase)	0.111 (0.090 to 0.133)	<0.001
Waist Circumference (1 cm increase)	0.008 (0.002 to 0.013)	0.007
IGFBP-3 [1 (AU) increase]	1.652 (0.989 to 2.314)	<0.001
**Alternative model (WHR instead of waist circumference at the stepwise algorithm)**		
PSA (1 ng/mL increase)	0.065 (0.033 to 0.097)	<0.001
WHR (1 numerical unit increase)	4.795 (2.560 to 7.030)	<0.001
IGFBP-3 [1 (AU) increase]	1.795 (1.204 to 2.386)	<0.001

Variables independently associated with log transformed total prostate volume.

Note: PSA, prostate specific antigen; IGFBP-3, insulin-like growth factor binding protein-3; WHR, waist to hip ratio.

Table 4 presents the results of the stepwise multivariate linear regression analysis regarding the volume of the TZ; interestingly, the stepwise algorithm resulted in the same independent predictor variables as in the case of TP volume. Specifically, higher PSA (coefficient: +0.148, 95%CI: +0.112 to +0.184, p<0.001), larger waist circumference (coefficient: +0.017, 95%CI: +0.009 to +0.025, p<0.001) and higher IGFBP-3 expression levels (coefficient: +1.019, 95%CI: +0.148 to +1.890, p=0.024) were independently associated with higher TZ volume. Once again, the alternative model (WHR instead of waist circumference) replicated the results.

**Table 4:** Results of the stepwise multivariate linear regression analysis.

**Variables Category or Increment**	**Coefficient (95% confidence interval)**	**P-value**
PSA (1 ng/mL increase)	0.148 (0.112 to 0.184)	<0.001
Waist Circumference (1 cm increase)	0.017 (0.009 to 0.025)	<0.001
IGFBP-3 [1 (AU) increase]	1.019 (0.148 to 1.890)	0.024
**Alternative model (WHR instead of waist circumference at the stepwise algorithm)**		
PSA (1 ng/mL increase)	0.100 (0.029 to 0.171)	0.008
WHR (1 numerical unit increase)	5.653 (1.104 to 10.202)	0.017
IGFBP-3 [1 (AU) increase]	1.072 (0.027 to 2.116)	0.045

Variables independently associated with log transformed transitional zone prostate volume.

Note: PSA, prostate specific antigen; IGFBP-3, insulin-like growth factor binding protein-3; WHR, waist to hip ratio.

## Discussion

Apart from their metabolic effects, IR and the counteractive increased insulin levels have also significant mitogenic and growth promoting effects that lay in the basis of the observed correlation between MS and BPH. Insulin itself is a well-known mitogen and growth factor [23], leading to prostate enlargement that also activates the IGF pathway, resulting in increased production of IGF-1 [24]. Most of the studies investigating the link between MS and its components with BPH, have examined serum concentrations of IGFs and IGFBPs although it is well accepted that serum levels of these markers may not reflect active intraprostate levels. Additionally [25,26], there is a lack of studies today focusing on the association between pre-diabetes, a condition also characterized by IR, with BPH and this is a field of great scientific interest given the high prevalence pre-diabetes in the general population [27]. 

In the present study we investigated the possible correlation between intraprostate expression of IGF-1, IGF-2 and IGFBP-3 with BPH, and according to our findings IGF-1 and IGF-2 levels of expression were not independent predictors of BPH. On the contrary, IGFBP-3 mRNA levels, waist circumference, as well as WHR, and PSA were found to significantly correlate with both TP and TZ volumes. 

Our findings are in accordance with the findings of Sarma et al., [3], that reported in a large scale study, that IGFBP-3 serum levels were independent predictors of prostate volume. Moreover, in the same study, no correlation was observed between IGF-1 serum levels and prostate volume. Colao et all., [28], have also reported that elevated IGFBP-3 levels correlate with prostate overgrowth among patients with acromegaly, a clinical syndrome which is also associated with hyperinsulinism, IR, overt diabetes and IGT, due to high serum GH and IGF-1 concentrations. In this context, our results further strengthen the above findings and expand their significance since we investigated intraprostate levels of expression of IGFBP-3 and IGF-1 that more accurately reflect what happens in situ. 

In contrast to our findings, other researchers have reported an inverse relationship between serum levels of IGFBP-3 with BPH [29,13]. However, it should be underlined that our results are not comparable with those of the previously reported studies since we investigated intraprostate levels of expression and not serum levels of IGFBP-3 or IGFs [29,13]. 

Today, IGFBP-3 is considered a protein with many pleomorphic actions, and not a simple carrier protein, regulating both proliferation and apoptosis in various cell types through its autocrine and paracrine actions. So, despite the well documented growth-inhibitory and apoptotic activity in many cell types, IGFBP-3 has been also associated with growth stimulation [30,31] in a variety of in vitro and in vivo models. IGFBP-3 can either [32] inhibit the mitogenic effect of IGFs by preventing IGF-1 from binding to its own receptor, or enhance IGF-1 actions by increasing IGF-1’s bioavailability at its receptor (IGF-1R). Given that most of the biological actions of IGFs on the fibromuscular stroma in BPH are mediated by IGF-IR [33], the proliferative effects of IGFB-3 on the fibromuscular stoma in BPH can be easily explained. In addition, overexpression of IGF-IR in various cell lines, as well as in prostatic stroma cells, results in reduced apotosis [34].

Experimental data demonstrated that pre-incubation of human fibroblasts with IGFBP-3, before the addition of IGF-I, was associated with accumulation of IGFBP-3 inside the cell, leading to specific forms of IGFBP-3, with lowered affinity for IGF-1 [35,36]. This facilitates a stable exchange of IGF-1 between the receptor and IGFBP-3 while avoiding of down regulation of IGF-1R by excess IGF-1 [35,36].

According to a recent study, the actions of IGFBP-3 are not predetermined and endogenous; IGFBP-3 is required for the action of both stimulatory and inhibitory factors within the same cell line [37]. 

An alternative pathway, by which IGFBP-3 contributes to BPH development in subjects with prediabetes, could be based on the relationship of adiponectin with BPH development [38-40]. Higher serum adiponectin is associated with marked reduction of BPH, but in contrast, adiponectin levels have been found to be reduced among prediabetics or obese subjects. Adiponectin levels are reduced even more since IGFBP-3 can inhibit adiponectin trancription [38-40]. 

Our findings regarding the observed no correlation between IGF-1 expression levels and BPH, are also in accordance with the findings of others concerning no correlation between IGF-1 serum levels and BPH [41,42]. Additionally, at univariate analysis, a correlation between levels of expression of IGF-2 and TP and TZ volume was observed, that lost its significance though at multivariate analysis. Interactions between IGFs and IGFBP-3 that regulate intraprostate IGF-1 and IGF-2 levels as well as IGF-1R levels most probably lay in the basis of the observed correlations and our findings further support the pivotal role of IGFBP-3 in the molecular pathophysiology of BPH.

Serum total PSA levels independently and positively correlated with TP and TZ volume according to our findings. PSA is a known protease that cleaves IGFBP-3. It also decreases the affinity of IGFBP-3 for IGF and can potentiate IGF action in the presence of inhibitory IGFBP-3 and contribute to normal and malignant prostate growth [43].

In our study, waist circumference and WHR positively and independently correlated with both TP and TZ volumes. These are in accordance with the findings of numerous epidemiological studies that have identified obesity in general and abdominal obesity, in particular as a significant risk factor for BPH [44,9]. IR, compensatory hyperinsulinemia and hormonal alterations associated with obesity, have been reported to be in the basis of the above [45].

Although ageing represents the central mechanism implicated in BPH development, recent novel findings also have highlighted the key role of the underlying age dependent hormonal alterations. The prostate enlarges with age in a hormonally dependent manner. These hormonal changes are driven essentially by obesity and in particular by abdominal obesity. In the ageing man, circulating levels of free estradiol remain constant due to an age-related increase in body weight and adipose cells. This imbalance between estrogen and testosterone levels, which has been implicated in BPH progress [46,47], is due to an increasing fat mass, which mainly accounts for the expression of high levels of aromatase [48], which generates increased estrogen production from peripheral conversion of androgens. 

Furthermore, it is well established that visceral fat accumulation, as expressed by waist circumference, is associated with IR and compensatory hyperinsulinemia [49], which has been associated with reduction in sex hormone binding globulin (SHBG), resulting to increase of the amount of androgen, entering the prostate cells, promoting to BPH development [50].

Regarding the relation of individual components of MS with BPH, we obtained similar results to Rohrmann et al. [51], who reported, using data from NHANES III, no significant relationship between lower urinary tract symptoms and total cholesterol:HDL ratio, LDL-C , triglycerides, or total cholesterol. Similarly, there was no association of patient-reported hyperlipidemia with histological BPH in a case control analysis on Italian men [52]. No association of serum lipids or lipoproteins with BPH was also found in a cohort of U.S. Air Force Veterans [53]. 

In contrast, Hammarsten et al. [4], found in a cohort of Swedish men with BPH, that lower HDL-C, higher LDL-C, and higher triglycerides were associated with increased prostate volume. These findings were in contrast to ours. In our study, with the exception of waist circumference, no association was seen among MS components and BPH, even though in univariate analysis, LDL-C and triglycerides were positively correlated with prostate volume (table 2). In addition, HDL-C correlated inversely with TP volume at a borderline significance. (r=-0.277, p=0.054). 

 However, lipid abnormalities are frequently related to the amount of visceral fat [54], and strong cross sectional associations [55] have been found among, waist circumference, an established measure of visceral adiposity and various metabolic risk factors, also implicated in BPH development. It can be assumed that waist circumference may have masked the effect of these metabolic risk factors on BPH development, because of these existing interrelations as our study suggests. 

As a final point, the lack of consensus on the exact definition of MS, the different populations, the existence of different end points, such as BPH or LUTS, make very difficult the direct comparison of study results. 

Although most of the studies demonstrated that MS might predispose patients to a higher risk for BPH, other studies, similarly to our findings, did not support this association [53,56-58]. However, it is well accepted that IR plays a major role in the pathophysiology of MS, even though NCEP criteria for identifying MS, does not include a marker of IR [59,60]. Since significant interrelations have been observed between abdominal obesity and IR [49], it seems reasonable that waist circumference, can be considered as a multivariate predictor of BPH, instead of MS presence, as shown by our study. 

There are several limitations to this study. First, serum concentrations of androgens and SHBG were not determined. However, we assumed that the results of our study were not significantly affected by the fact that androgen or SHBG levels were not included among the adjusting co-variates, given that androgen action is mainly indirect through prostatic production of some growth factors [61]. As men age, the concentration of free testosterone decreases, but dihydrotestosterone (DHT), its intracellular metabolite, generated by the prostatic 5-a reductase [62] continues to accumulate and stimulates production and secretion of growth factors, promoting the growth of cells. DHT binds with androgen receptors and triggers the transcription of growth factors, which in turn cause prostatic tissue growth. Thus, the effect of both testosterone and DHT on prostate volume is carried by growth factors [63,64,65]. 

Second, the cross-sectional design of our study does not allow establishing cause-effect relationship. 

Third, homeostatic model assessment (HOMA) IR was not calculated. Despite the wide use of HOMA-IR, HOMA IR is not an ideal way to measure IR, given that there is no consensus for HOMA-IR cut off values for identifying subjects with IR. In view of the fact that IR and abdominal obesity are positively related [49,66] and the risk of IR is positively associated with increasing waist circumference [67,68], HOMA –IR finally was not finally determined in our study and waist circumference was determined as a proxy index for insulin resistance [69]. At the end, it is well accepted that waist circumference provides a rapid, inexpensive and non-invasive way of identifying the presence of IR [70].

Fourth we could not rule out confounding by variables such as physical activity, smoking, alcohol consumption, since our multivariate models were not adjusted for these variables. 

In summary, our findings show that intra-prostatic levels of IGFBP-3, PSA and waist circumference, but not overall obesity, are positively associated with prostate volume. IGFBP-3 seems to be a multifunctional protein, which can potentiate or inhibit IGF activity. However, in a dynamic in vivo system, homeostatic mechanisms cause compensatory responses and form complex interrelations, thus it becomes extremely difficult to estimate the independent effects of each protein implicated in BPH pathogenesis. To the best of our knowledge, this is the first study to demonstrate a relationship between intra-prostatic levels of IGFBP-3 and prostate volume in subjects with pre-diabetes. Future investigation is needed to further elucidate the relationship between obesity, glucose intolerance and IGF-IGFBP- system and prostatic growth, targeting to new treatment strategies focused on diet, exercise, and drugs inhibiting prostate cell proliferation. 
